# Dynamic sensitivity analysis: Defining personalised strategies to drive brain state transitions via whole brain modelling

**DOI:** 10.1016/j.csbj.2022.11.060

**Published:** 2022-12-01

**Authors:** Jakub Vohryzek, Joana Cabral, Francesca Castaldo, Yonatan Sanz-Perl, Louis-David Lord, Henrique M. Fernandes, Vladimir Litvak, Morten L. Kringelbach, Gustavo Deco

**Affiliations:** a Centre for Brain and Cognition, Computational Neuroscience Group, Department of Information and Communication Technologies, Universitat Pompeu Fabra, Barcelona, Spain; b Centre for Eudaimonia and Human Flourishing, Linacre College, University of Oxford, UK; c Life and Health Sciences Research Institute (ICVS), School of Medicine, University of Minho, Braga, Portugal; d Centre for Music in the Brain, Aarhus University, Aarhus, Denmark; e Department of Psychiatry, University of Oxford, Oxford, UK; f Institució Catalana de la Recerca i Estudis Avançats (ICREA), Barcelona, Spain; g Department of Neuropsychology, Max Planck Institute for Human Cognitive and Brain Sciences, Leipzig, Germany; h School of Psychological Sciences, Monash University, Melbourne, Australia; i Wellcome Centre for Human Neuroimaging, University College London, Queen Square Institute of Neurology, London, UK; j National Scientific and Technical Research Council (CONICET), Buenos Aires, Argentina

**Keywords:** Spatio-temporal dynamics, Brain stimulation, Whole-brain models, Brain State, Magnetic Resonance Imaging, MRI, functional Magnetic Resonance Imaging, fMRI, diffusion Magnetic Resonance Imaging, dMRI, Position Emission Tomography, PET, Non-Invasive Brain Stimulations, NIBS, transcranial Electric Stimulation, tES, transcranial Direct Stimulation, tDCS, transcranial Alternating Current Stimulation, tACS, transcranial Random Noise Stimulation, tRNS, Transcranial Magnetic Stimulation, TMS, Deep Brain Stimulation, DBS, static Functional Connectivity, sFC, dynamic Functional Connectivity, dFC, Probability Metastable Substates, PMS, Transition Probability Matrix, TPM

## Abstract

Traditionally, in neuroimaging, model-free analyses are used to find significant differences between brain states via signal detection theory. Depending on the a priori assumptions about the underlying data, different spatio-temporal features can be analysed. Alternatively, model-based techniques infer features from the data and compare significance from model parameters. However, to assess transitions from one brain state to another remains a challenge in current paradigms. Here, we introduce a “Dynamic Sensitivity Analysis” framework that quantifies transitions between brain states in terms of stimulation ability to rebalance spatio-temporal brain activity towards a target state such as healthy brain dynamics. In practice, it means building a whole-brain model fitted to the spatio-temporal description of brain dynamics, and applying systematic stimulations in-silico to assess the optimal strategy to drive brain dynamics towards a target state. Further, we show how Dynamic Sensitivity Analysis extends to various brain stimulation paradigms, ultimately contributing to improving the efficacy of personalised clinical interventions.

## Graphical abstract

**Figure:**
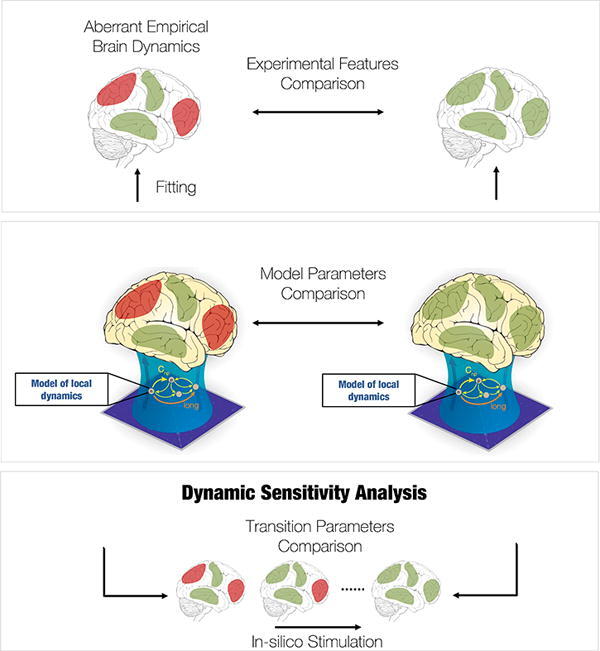

## 1 Introduction

Over the last century, the search for therapeutic strategies to re-establish mental health has relied on clinical trials. Despite the ethical constraints of human experiments - and in particular of vulnerable psychiatric populations -, this trial-and-error approach has led to the discovery of therapies driving transitions from diseased brain states towards a healthy brain state, using pharmacotherapy, electromagnetic pulses or even by self-regulating behaviour [1], [2]. However, the efficacy of these strategies remains suboptimal and the pace of development is slow, mainly due to the high variability of treatment outcomes and a lack of computational tools to assist in the design of personalised therapeutic strategies [3].

In neuroimaging, many studies serve to reveal statistically significant differences in brain activity in different disorders via signal detection theory, or more colloquially, p-value testing [4], [5]. In particular, the activity recorded with functional Magnetic Resonance Imaging (fMRI) at rest - a task-free condition - reveals alterations in spatial and temporal features of macroscopic (whole-brain) activity patterns. More specifically, differences in terms of regional activations, functional connectivity and fractional occupancy of dynamic state-based approaches provide quantifiable distinguishing features associated with specific brain disorders [6], [7], [8]. Yet, it is unclear what underlying mechanisms give rise to the observed differences.

Over the last decade, whole-brain network models have served to investigate possible mechanistic scenarios for the origin of the differences detected at the empirical level with interpretations at the level of changes to the local/global coupling strength, alterations in the local dynamics (gain modulation/stability of oscillations) [9], [10], [11]. We further elaborate on whole brain computational models in the section “Brain networks models”. However, these models serve mostly to address the pathophysiology of mental disorders by studying the model parameter changes and do not aim at finding causal ways of transiting between various brain states with the consequences for the treatment of disorders.

Here, we propose Dynamic Sensitivity Analysis as a general framework to make computer-assisted predictions of optimal stimulation strategies to rebalance spatio-temporal dynamics between brain states using whole-brain network models. So in this perspective “to rebalance” means to drive the brain’s dynamical repertoire to that of the dynamic range associated with healthy neurotypical brain states. In the context of Dynamic Sensitivity Analysis, we would for example consider the brain dynamics to be re-balanced if they achieve the same statistical distribution of Probability Metastable Substates (PMS) as the control group / condition. It builds on a paradigm initially proposed by Deco and colleagues to investigate the brain regions more prone to drive transition between awake and asleep states [12]. The same paradigm was recently extended to the clinical context for treatment-resistant depression, to find the brain regions that work in promoting a transition to a target healthy state in responders but not non-responders to treatment with the psychoactive compound psilocybin [13]. The approach revealed a map of brain regions that significantly overlaps with the density map of specific serotonin receptors (5HT_2A_ and 5HT_1A_) to which psilocybin is known to bind preferentially [14], corroborating the hypothesis that the therapeutic effects of psilocybin are linked to a modulation of the serotonergic system. Another study in the ageing population of older participants showed region-specific stimulations that approximate middle-aged population brain dynamics [15]. Being free from ethical limitations, such in-silico approaches provide new means to systematically investigate the impact of distinct stimulation strategies on brain dynamics [16], [17] and evaluate their ability to drive transitions to a desired dynamical brain state [12]. Here, we describe how the framework can be extended to a wide array of stimulation types, ranging from pharmacological to electromagnetic (such as TMS, tES and DBS), which can be applied either at a single focus or distributed across the brain, in order to mimic realistic interventions and bring about personalised modelling strategies for recovery in various brain disorders.

Here, we focus on brain state transition from the perspective of Dynamic Sensitivity Analysis. However, related approaches have been considered. For example, control network theory has been used to force transitions in large-scale brain networks. In this scenario, different control strategies are used to navigate brain dynamics from a source to a target brain state [18]. Control theory, due to its wide ranging applicability in technological, social and cyberphysical systems across various experimental scenarios, has received a lot of attention [19], [20]. Unlike in Dynamic Sensitivity Analysis where the focus is on rebalancing the spatio-temporal brain dynamics, control theory focuses on “controlling” the temporal trajectory between various source and target states.

## 2 Spatio-temporal dynamics of brain states

The contention here is that Dynamic Sensitivity Analysis can be a useful tool to describe and evaluate strategies to transition between various brain states. In this direction, an important aspect is to accurately define what is understood as a brain state. Behaviourally, a brain state can be described by the condition in which an individual is engaged, ranging from a task-free resting state, to passive music listening or movie watching, or while performing an attentional or cognitive task, or even sleeping in a given sleep stage. Altered brain states can be induced by psychoactive drugs, such as psychedelics, or appear in clinical context due to underlying psychiatric conditions, such as schizophrenia, major depressive disorder and autism, or neurological diseases such as Alzheimer’s disease, or disorders of consciousness. Yet, at the biophysical level, a more objective view is to define a brain state as a dynamical regime in which the brain - as a collective system - exhibits characteristic activity patterns defined in space, time and frequency domains, which are accompanied by characteristic behavioural correlates [21]. This implies that distinct brain states can be discerned by intrinsic differences in their spatio-temporal dynamics. Importantly, unlike event-related signals, which consist in the response functions triggered by a perturbation/stimulus/task/event, the metrics characterising a brain state are expected to exhibit stationarity within a brain state.

**Fig. 1:**
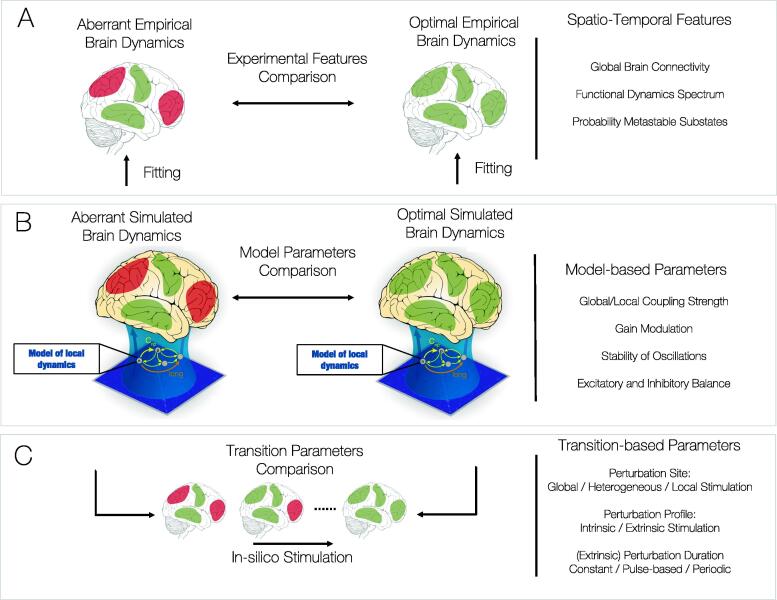
Conceptual Overview of Dynamic Sensitivity Analysis A) Descriptive Analysis. Traditional statistical analysis between empirical fMRI brain recordings of different study groups (example control and patient groups). Dynamics can be described by various features across space and time, for example Global Brain Connectivity, Functional Dynamics Spectrum or Probability Metastable Substates (PMS). B) Explanatory Analysis. Generative modelling approaches to describe the emergent dynamics of coupled dynamical units in the brain network. Network models can be adjusted to approximate spatio-temporal features of brain dynamics at the individual or group-level by tuning model parameters relating to various brain mechanisms, such as global/local coupling strength, gain modulation, stability of oscillations or excitatory/inhibitory balance. Relating to different brain mechanisms, these parameters can subsequently be statistically compared between conditions to obtain model-based features characteristic of each brain state. C) Predictive Analysis. The framework of Dynamic Sensitivity Analysis consists in the systematic investigation of the optimal strategy promoting a transition between distinct brain states. This framework provides a non-invasive means to evaluate the nonlinear response of distinct perturbation strategies aimed at promoting a transition from an aberrant brain state to an optimal and healthy brain state.

Over the last century, the neuroimaging community has collected an evermore detailed description of brain activity patterns associated with distinct brain states. For instance, the slowing down of oscillations detected with electroencephalography upon falling asleep is one of the first descriptions of a robust physical correlate of a brain state transition [22]. More recently, with the shift of fMRI studies away from event-related responses towards ‘task-free’ recordings, novel features characterising brain states have been revealed. However, it is to be noted that how complexity at microscale impacts the assumptions of global brain states sustained in time is not trivial. Using network-based approaches, a growing literature has shown that brain activity in various brain states can be described in terms of long-range correlations of ultra-slow fluctuations (0.01–0.1 Hz) detected in segregated brain areas, shaping state-specific functional connections and networks [7], [23], [24]. Initially, these network-based metrics considered functional connections to remain stable within a brain state, and henceforth correlations were computed over the longest possible observation time within a brain state, which is now termed static functional connectivity (sFC). Only over the last decade, the temporal aspects of the brain's interplay between areas, generally termed dynamic Functional Connectivity (dFC), have been explored with various techniques [8], [25]. Yet, it is unclear what the best description of the brain's complex dynamics is. Many methods have been developed to study dynamic functional connectivity [21]. Often they can be summarised in terms of quantification of signal intensity or variability of different brain regions, as a temporal trajectory in a landscape of spatial substates and spatio-temporal graphs [26], [27], [28]. Accordingly, various features can be extracted from these approaches: signal/connectivity diversity in time or between individual brain regions, a description of time-varying spatial substates in terms of probability of occurrence, life times and probability of transitions, and graph theoretical metrics with temporal dimension. Such rich spatio-temporal description has been shown to distinguish many brain states (not their transitions) [29], [30], [31], [32], [33] and serves as a first step in the Dynamic Sensitivity Analysis pipeline (Fig. 1A).

The appropriate choice of spatio-temporal features in any Dynamical Sensitivity Analysis should consider how reliable and specific the studied features are. In particular, in clinical settings the stability of measures across scanning sessions and the ability to distinguish adequately between subjects is of great importance [34], [35]. In line, fingerprinting based on functional and dynamic connectomes has shown to be specific and identifiable [36], [37], [38]. Furthermore, dynamic descriptions of time varying substates have shown reliability across scanning sessions and subjects [27]. Additionally, models fitted to the static FC performed well in terms of reliability and specificity, especially when enhanced model personalisation was considered [39]. In other words, understanding the reliability and specificity of spatio-temporal features enforces further steps of Dynamic Sensitivity Analysis.

## 3 Brain network models

Theoretical and computational neuroscience aim to develop and search for mechanistic models that explain the empirical observations of neural activity recorded across modalities and across scales [40], [41]. Although initially focused on the microscopic scale of neurons and neuronal pools, there has been a growing interest in extending computational models of neuronal activity to the macroscopic whole-brain level in order to address the mechanisms that give rise to the emergent spatio-temporal whole-brain dynamics. Given the growing hypothesis that the principles orchestrating brain activity at the macroscopic scale are related to interactions of neural activity in the neuroanatomical network, the general rationale is to use reduced models to mimic the emergent spatio-temporal brain dynamics as an interaction of non-linear local dynamics, approximating the collective dynamics of neuronal pools, connected through a network of biologically plausible topology [9], [10], [11], [42], [43]. It is important to note that linear whole-brain models have also been fitted to the functional MRI signals, such as multivariate Ornstein-Uhlenbeck process or spatial autoregressive (SAR) models, with success and sometimes outperforming their non-linear counterparts [44], [45], [46], [47]. However, there is growing evidence that they fall short of reproducing the spatio-temporal features of brain dynamics [48]. Following different reduction lines, several models have been put forward to simulate the spontaneous dynamics of brain networks. In general, brain network models consist of a set of coupled differential equations with assumptions made at the level of neuronal physiology or phenomenological dynamics. Often, the choice of the local model representing the dynamics of a brain region depends on an experimental question at hand and a delicate balance between modelling of biophysical realism and complexity [39], [49]. Specifically, the local mean field of neuronal activity can be approximated considering the mean membrane potentials of a population of neurons taking into account the conductance and Nernst potentials of different ions and/or the ratio between excitatory and inhibitory neurons [42], [50] on one hand, and phenomenological descriptions of dynamic profiles, such as phase- or phase-amplitude- oscillators on the other [51], [52].

Given the set-up of whole-brain models, there are several features one can optimise in order to arrive at a mechanistic interpretation of a brain state (Fig. 1B). Below we provide a brief description of the main elements of a brain network model, namely the connectivity structure, the local dynamics, the coupling functions and the heterogeneity, and comment on the implications in brain network dynamics.

### 3.1 Connectivity

Different brain regions are connected through a dense network of axonal projections, which can be captured non-invasively using tractography algorithms applied to diffusion MRI (dMRI). These tractograms have been validated through ex-vivo track-tracing in non-human mammals, and provide a close approximation of the large-scale wiring diagram of brain structural connectivity - the Connectome [53], [54]. The Connectome serves to define the structural scaffold of brain network models, with the number of tracts detected between a pair of brain regions being used to scale the relative coupling strength between units in the network. While the connectome constrains the topology on which the spatio-temporal dynamics emerge, it remains mostly invariant over the timescales of brain recordings. From a perspective of brain states, it is assumed to remain unchanged when a subject transits from one brain state to another over relatively short time intervals, such as when falling asleep or entering a psychedelic state, and therefore additional model parameters are necessary to explain brain states and their differences. Still, it is believed that the structural Connectome may be affected in certain neurological disorders where structural lesions are detected at the level of white matter between patients and healthy controls [55], [56].

### 3.2 Local-dynamics

Parameters related to local neuronal dynamics of a brain area - representing a node in the brain network model - depend on the type of reduced mesoscopic models chosen for analysis. In biophysical models, where the mesoscopic activity emerges from the balanced interaction between excitatory and inhibitory pools of neurons (for example the mean field or Wilson-Cowan models), the coupling weights are tuned to describe the level of influence different pools of neurons have on each other [57], [58]. Similarly, the gain function transforming the incoming synaptic current into a firing rate is related to the excitability of neuronal populations and can be also modulated [59], [60]. In phenomenological models such as the ones describing the oscillatory response of a neuronal pool, parameters of the local node include the natural frequency and, when the amplitude dynamics is considered (such as in the Stuart-Landau equation), a bifurcation parameter can be tuned to modulate the stability of the oscillations [61], [62].

### 3.3 Coupling functions

The term of how the activity in one node is affected by the activity in the other nodes is scaled by a global coupling parameter, that must be tuned at the right balance to replicate the non-steady dynamics observed in brain activity [50], [63]. In addition, the relative coupling between each pair of units is scaled by the structural connectome but can also be tuned to fit the directed connectivity estimates across various brain states, assuming a dynamic component modulating the coupling at the synaptic level [46], [64]. Beyond the strength of coupling, another important ingredient in the model is the interaction function, i.e., how one unit responds to the activity in the others [65]. Again, different scenarios have been proposed, ranging from linear diffusive coupling between excitatory pools [50], to phase synchronisation between oscillators based on the Kuramoto model, or even coupling both the amplitude and the phase of the complex analytic signal [62], [63].

### 3.4 Heterogeneity

Another important aspect of brain organisation is its heterogeneous anatomical, histological and cellular properties. Using PET imaging or distinct MR pulse sequences, studies have shown maps of heterogeneity in cortical and subcortical regions in terms of neuroreceptor density maps [66], gene expression [67], temporal time-scales [68], myelin content as indexed by T1/T2-weighted MRI signal [69] and functional connectivity [70]. Tuning models with heterogeneous information about molecular and cellular composition further extends the broad dynamic range of possible emergent dynamics [59], [71], [72]. Such heterogeneity maps may be obtained at the individual level to inform personalised models on which the inference is based [59].

## 4 Dynamic Sensitivity Analysis (Modelling Stimulations)

A brain state can be characterised in terms of its spatio-temporal dynamics derived from the data or inferred from the mechanistic parameters of the brain network models, as discussed in the previous two sections. Yet, the proclivity of transition to another state is an important feature in the characterisation of brain states (Fig. 1C). For instance, reduced states of consciousness such as sleep, disorders of consciousness and unresponsive wakefulness state can be understood in terms of their susceptibility/resilience to perturbation and thus can be characterised with different metrics of stability and reversibility [73]. Similarly, perturbation analysis has shown path dependency when transiting from and into a sleep state [12], [74].

**Fig. 2:**
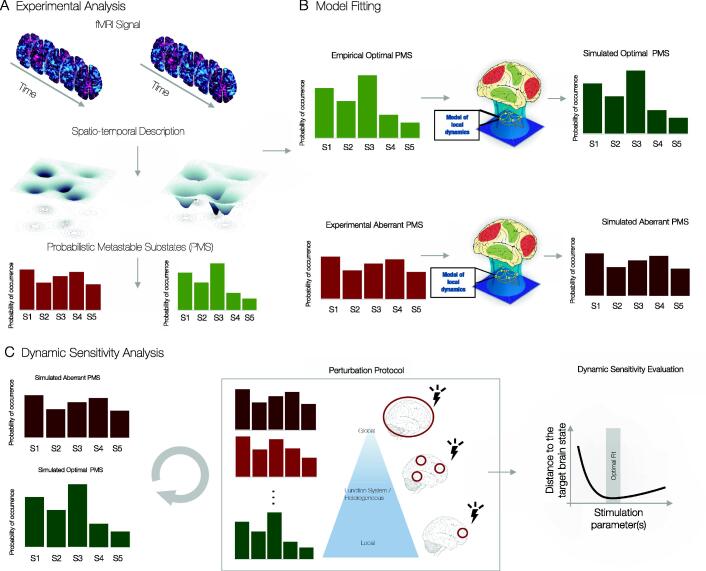
Design Overview of Dynamic Sensitivity Analysis A) Experimental Analysis. fMRI signal is converted into a spatio-temporal description. Here, we focus on the Probability Metastable Substates (PMS) as a way to summarise brain dynamics across the spatial and temporal dimension. B) Model Fitting. Whole-brain models for optimal and aberrant dynamics are optimised to the PMS. C) Dynamic Sensitivity Analysis. An optimal transition to the target state is systematically explored by applying a perturbation protocol with varying parameters. D) Dynamic Sensitivity Analysis Evaluation. Varying perturbation sites, profiles, time durations and intensities are explored and evaluated for the optimal fit to the target description of PMS.

In practice, this means describing brain states with various spatio-temporal features. Fig. 2A shows how spatio-temporal dynamics can be described as a temporal trajectory of brain states in a landscape of spatially relevant attractors. By doing so, a brain state can be characterised in terms of the probability of occurrence of the individual substates, termed Probability Metastable Substates or other summary statistics measures describing spatio-temporal brain dynamics. The error minimization of these experimental features is used as an objective function in the fitting step where a range of model parameters is tuned to approximate the model and experimental dynamics. For example, the Kullback-Leibler distance is considered to quantify the similarity of the simulated and experimental PMS features. Then, the correct parameter choice of the whole-brain model is used to approximate the different brain states (Fig. 2B). In turn, this is then used to perform an in-silico perturbation protocol to study the effects of various stimulations on the unfolding spatio-temporal dynamics (Fig. 2C).

**Fig. 3:**
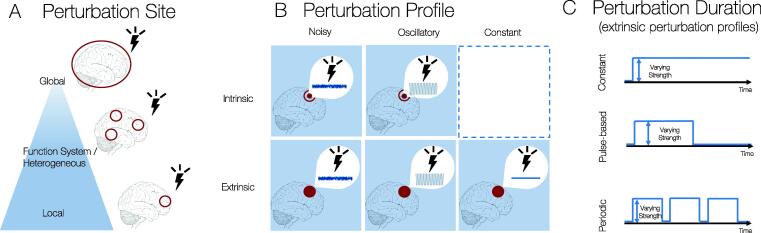
Types of Perturbation Protocols. A) Perturbation Site: Stimulations to promote brain transitions can be applied at the global level to all regions of interest, at the mesoscopic level defined by functional systems or other maps defining heterogeneity in space such as neurotransmitter receptor maps, T1/T2 weighting or transcriptomics gradients or at the local level for individual brain regions. B) Perturbation Profile: Stimulations to promote brain transitions can reflect intrinsic (changes in local brain dynamics such as the bifurcation parameter in Hopf-model and the gain function in Wilson-Cowan model) and extrinsic effects (modelled via an additional term reflecting the stimulation). Stimulations can be noisy, oscillatory or constant. C) Perturbation Duration: Stimulations for the extrinsic perturbation profiles can reflect on-going, constant, pulse-based short-lived or periodic effects.

The stimulation protocol can be explored exhaustively using deep learning algorithms or in a hypothesis-driven manner. Previously defined hypotheses may constrain the range of parameters explored but also constrain the range of possibilities discovered. For instance, it is possible to define a priori the type of stimulation applied (i.e., an increase in excitation, a change in frequency or adding a sinusoidal pulse, among others), the location where the stimulation is applied (i.e, locally or globally, homogeneously or heterogeneously). In terms of heterogeneous stimulation, this may be constrained by targeting specific functional subsystems associated with the density of neuroreceptor maps, the neuroanatomy or topology of the connectome (Fig. 3, A). The profile of stimulations may be intrinsic or extrinsic. Internally driven stimulations will depend on the mesoscopic model choice of the local dynamics. Often, this will result in a combination of noise-induced and oscillation-induced dynamics. Alternatively, an external perturbation can be introduced as an additional term in the equations to drive the mesoscopic model description simulating a combination of noisy, oscillatory and constant external inputs (Fig. 3B). Furthermore, the duration of perturbation can be specified from a constant (long-term) to pulse-based and periodic oscillations (Fig. 3C). Furthemore, Table 1 describes examples of the types of properties that can be affected by stimulations, how they can be modelled mechanistically and suggestions for relevant model choices.

**Table 1:** Modelling Examples for Brain Stimulations. Spiking Neurons - SN [75], Fitzhugh-Nagumo - FN [76], Jansen-Ritt - JR [77], Conductance-Based Biophysical Model - CBB [78], Dynamic Mean Field - DMM [79], Wilson-Cowan - WC [50], Stuart-Landau - SL [63], Kuramoto Model - KM [51], Exact Mean Field Model - EMFM [80].

Property affected by perturbation	Model Parameters that can be modulated	Model examples
Firing Rate	Shape of the transfer function from input current to firing rate (i.e., as a function of receptor density such as 5HT_2A_)	DMM, WC, EMFM, JR
Excitation and Inhibition	Ratio of excitatory and inhibitory neurons	SN, CBB, WC, DMM, EMFM
	Weights of excitation-to-inhibition and recurrent excitation	SN, CBB, WC, EMFM, JR
Local Field Oscillations	Frequency	SN, KM, FN, WC, SL
	Stability, Amplitude, Ratio between noise and oscillations	SN, SL

## 5 Case studies

### 5.1 Neurophysiological stimulation

Perhaps one of the most obvious brain state transitions is that of falling into sleep. It is a natural physiological process happening every night and serves as a great example to demonstrate the fecundity of Dynamic Sensitivity Analysis. It has been shown that the brain follows a specific dynamic choreography of brain substates while moving through the wake-non-REM sleep cycle [74]. Interestingly, this happens with hysteresis where the trajectory between transitions to falling asleep are different to that of waking up. In this context, sleep and wakefulness brain states have quantitatively different dynamic stability to perturbations [73] and Dynamic Sensitivity Analysis can serve to probe the physiological mechanism that “puts us to sleep at night and wake us up in the morning”.

To understand the transition between wakefulness and sleep brain states Deco and colleagues used principles of Dynamical Sensitivity Analysis to explore the regions responsible for driving the transitions between the two states [12]. First, they described the fMRI activity of both brain states in terms of the Probability Metastable Substates and Transition Probability Matrices (TPM). This has been achieved by using an unsupervised clustering algorithm on the leading eigenvectors, derived from the instantaneous phase coherence matrices at every time point [27], [32]. The number of clusters was chosen to be three according to various criteria such as the Silhouette score and the smallest number of states to statistically separate the two brain states. Then, the authors proceeded to build whole brain models (Hopf model) of wakefulness and sleep by optimising the Global coupling parameter G and Effective Connectivity, demonstrating an optimal fit to the PMS and TPM features. Furthermore, systematic perturbation of homotopic (contra-lateral) connections was carried out by varying the bifurcation parameter between noisy and oscillatory regime to rebalance the dynamics between the two states. The authors showed that it was possible to “awaken” the brain from sleep with weaker oscillatory activity compared to the other way around where stronger noisy stimulation was required to bring the dynamics to “sleep”. Moreover, they further showed that weaker multi-site perturbation could achieve similar outcomes when driving sleep brain state to that of wakefulness. These results motivate further Dynamical Sensitivity Analysis studies where more physiologically-inspired whole-brain models can give further probe into the biological mechanism leading to the wake-non-REM sleep cycle.

### 5.2 Neuropharmacological stimulation

One of the objectives of translational neuroscience is to predict the effects of pharmacological drugs on emergent spatio-temporal dynamics and thus propose recovery response for various brain disorders. In psychiatry, there has been a growing interest in psychedelic medicine for conditions such as depression, addiction and anorexia. One of the leading hypotheses is that administration of psychedelic drugs together with psychological interventions provide a window of malleability whereby negative cognitive biases and associated ruminative thoughts can be reassessed [81]. This has been demonstrated with acute effects of psychedelics, suggesting an increase in the repertoire of functional substates [82], [83], [84]. On the pharmacological level, psychedelics are known to bind with high affinity to the serotonergic receptors, especially the 5HT_2A_, but other receptors play a part, and by doing so modulating the excitatory-inhibitory balance towards more excitation [14], [85]. Recent whole-brain modelling work has demonstrated the causal link between the impact of 5HT_2A_ agonist-induced excitation on the spatio-temporal dynamics [59].

Assessing how the intervention with psychedelics can promote transition between pathological and healthy brain dynamics has been explored in recent work where intrinsic perturbation was exhaustively performed to demonstrate regional effects on promoting transition from aberrant to optimal brain dynamics [13]. First, the authors analysed different features of spatio-temporal dynamics, such as Global Brain Connectivity, Metastability, Synchrony, Functional Connectivity Dynamics and Probabilistic Metastable Substates, demonstrating the importance of the temporal dimension in separating responders from non-responders after the treatment. The differences in the PMS served as the basis for construction of two whole-brain models (in this case modelled as a network of coupled Hopf oscillators) fitted by adjusting the global coupling parameter to the PMS of responders and non-responders before the treatment. The PMS description of the responders after the treatment served as a target working point against which the subsequent stimulation analysis was quantified. Then, the stimulation protocol was implemented by independently stimulating homotopic (contra-lateral) regions and comparing the resulting PMS distribution to that of the target state. Here, the authors used an intrinsic stimulation paradigm in which the brain regions can exhibit a more noisy or a more oscillatory behaviour depending on the value of the bifurcation parameter of the Hopf model representing each brain region. By exhaustive stimulation the authors have demonstrated that there is a subset of regions that are more prone to drive a rebalancing of the PMS distribution to the target post-treatment PMS for responders but not-nonresponders. Moreover, the proclivity to this rebalancing effect negatively correlates with the 5HT_2__A_ and 5HT_1__A_ serotonergic receptor density maps from healthy subjects, suggesting that the impact of pharmacological stimulation in brain dynamics is directly related to the distribution of specific serotonin receptors across distinct brain structures. For future work, knowing the pharmacology of psychedelic drugs, Dynamic Sensitivity Analysis could explore how different 5HT heterogeneity maps are relevant for the transitions between states.

### 5.3 Invasive brain stimulation

Deep brain stimulation (DBS) is a powerful neurological procedure used in the treatment and control of many neurological and psychiatric disorders such as Parkinson’s disease (PD), dystonia, essential tremor (ET), drug-resistant epilepsy and obsessive–compulsive disorder (OCD), which aims at disrupting the abnormal oscillatory neural activity [86], [87]. It consists in implanting electrodes into specific brain structures and delivering high-frequency constant or intermittent current from a subcutaneously implanted pulse generator [86].

The response time to the treatment varies incredibly depending on the disorders and its associated symptoms in question, suggesting that DBS may act through different mechanisms going from immediate network neuromodulation (rapid-response symptoms such as tremor, rigidity for PD or anxiety for OCD) to synaptic plasticity and anatomical remodelling (slow-response symptoms such as axial symptoms in PD or mood in OCD) [88].

The mechanisms by which high-frequency stimulation ensures a decrease in symptoms is still a matter of debate. Evidence supports the hypothesis that either direct stimulation within Globus Pallidus internus (GPi) or the Subthalamic Nucleus (STN), reduces the GPi inhibitory output neurons activity and therefore increases the thalamic target neurons activity, remarking the inhibitory role of DBS. The local inhibition may be caused by various processes, for example the inactivation of voltage-gated currents [89], activation of inhibitory afferents [90] and sustained depolarization of neuronal cell membranes [91]. Conversely, evidence also supports the excitatory effect of DBS, for example when stimulating the STN neurons, the GPi, Globus Pallidus externa (GPe), Substantia Nigra (SNr) firing rates increase [92]. Additionally, changes also occur from the neurochemical perspective. Once action potentials are generated, they propagate to the axon terminals and induce neurotransmitter release both locally and throughout the network of interest. The DBS-induced changes depend on the specific microcircuit stimulated and the effect can be either excitatory (via modulated glutamate release) or inhibitory (via modulated GABA release).

On this note, a correct approach to underpin DBS mechanisms will need to contemplate the empirical structural and functional patterns, as well as underlying neurochemical alterations – spanning across pathways, microcircuits, and whole-brain levels. Computational models have been able to describe empirical features as well as to predict local activation patterns and transition between states [88], [93], [94], [95]. This could not only provide a comprehensive empirical framework for interpreting how DBS is reorganising brain diseased circuitry but could ultimately serve to improve the precision of surgical planifications and risk assessment, and consequently improve the success rate of DBS interventions [96].

### 5.4 Non-invasive stimulation

Over the last three decades non-invasive brain stimulations (NIBS) such as transcranial electric stimulation (tES) and transcranial Magnetic stimulation (TMS) have been shown as valuable options aas treatments for psychiatric and neurodegenerative brain disorders such as depression, epilepsy and dementia [97] as well as disorders of consciousness [98]​​. NIBS tools work by delivering weak electric currents through the scalp and thus modulating brain function by interacting with neuronal tissue. Such stimulation profiles can vary from alternating currents (transcranial Alternating Current Stimulation (tACS)), noisy (transcranial Random Noise Stimulation (tRNS)), direct currents (transcranial Direct Stimulation (tDCS)) or supra-threshold intracranial electric field pulses evoked by TMS. In all instances, the current stimulation modulates how information is processed within neuronal tissue leading to top-down plasticity changes in terms of long-term potentiation (LTP) and long-term depression (ltd) [99].

Alzheimer’s disease is a neurodegenerative disease causing a decline of cognitive functioning that is worsening throughout years with no disease-modifying therapies available, and for which pharmacological therapies provide only modest symptomatic improvements. While the neurophysiological causes of Alzheimer’s disease are still debated, one of the main predictors of the onset and cognitive deterioration is the accumulation of protein aggregates: amyloid-beta (Aβ) and phosphorylated tau protein (p-tau). Another hallmark of the disease progression is the slowing of brain oscillatory activity and particularly of the alpha rhythm (8–12 Hz). In terms of network effects, the cascading network failure model has suggested that an aberrant hyper-synchronisation at early AD stages precedes the hypo-synchronisation at later AD stages [100].

A seminal paper in a mouse model has demonstrated a reduction of Aβ plaques after a 40 Hz tACS stimulation. In turn, this has been shown to halt subsequent neuronal degeneration and behavioural impairments, promising a powerful therapeutic intervention and paving a way for human clinical trials [101]. A recent intervention-based human clinical trial has demonstrated the feasibility of repeated sessions of 40 Hz tACS with different scalp montages in mild to moderate AD patients leading to promising outcomes in regulation of cerebral perfusion, spectral power changes in gamma band (∼40 Hz) as well as cognitive performance [102]. However, how the underlying spatio-temporal dynamics changes can be described, explained and predicted through the repeated 40 Hz tACS stimulation is still unknown and deserves to be explored through Dynamic Sensitivity Analysis as argued for in this review.

## 6 Discussion

**Fig. 4:**
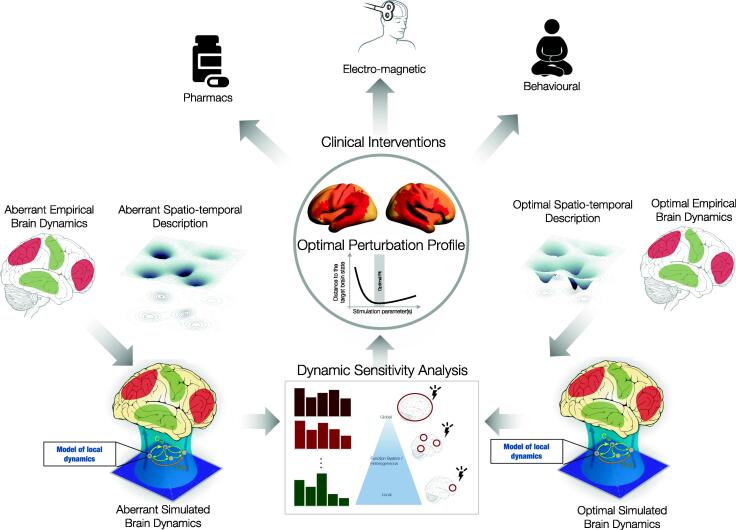
Personalised in-silico brain model to predict therapeutic outcomes. The principles of Dynamic Sensitivity Analysis using whole-brain computational models open up for novel clinical intervention design. The schematic provides a pipeline for how to combine functional information about the brain spatio-temporal dynamics in a model to describe the aberrant brain state (leftmost). Once the model is optimised, exhaustive Dynamic Sensitivity Analysis can be performed to establish perturbation sites, profiles and/or durations for optimally rebalancing the aberrant dynamics towards a target brain state. Lastly, based on the optimal perturbation profile, clinical interventions can be suggested that are most compatible with the suggested in-silico intervention.

In this review, we have argued that Dynamic Sensitivity Analysis might deliver in-silico solutions for describing transitions between brain states either in an exhaustive or hypothesis-driven way and thus paving direction to a personalised strategies in health and disease. In translational neuroscience this would mean devising in-silico perturbation strategies to an individual patient/condition and through the suggested perturbative profile offering different clinical interventions (Fig. 4). In doing so, further progress will require 1) a clear spatio-temporal description of brains states, 2) whole-brain computational models providing strongly representative descriptions of the empirical brain dynamics at the individual level, 3) in-silico testing of the various perturbative strategies and 4) further experimental studies proving/disproving the suggested outcomes of the perturbational profiles and capturing/approximating the real response function. Moreover, a general consideration and future challenge for successful personalised/precision in-silico interventions for clinical treatment is that of sensitivity and reliability. Recent work has shown that whole-brain models are sensitive and reliable when fitted to the individual static features of brain dynamics, especially with enhanced model personalisation [39]. Further work should as well explore a similar study paradigm in the dynamical measures. Lastly, another important aspect of successful interventions will be to first quantify the dimension of neuronal processing and thus choose whole-brain models and their free parameters accordingly. This will allow for adequate determination of how complex the individual brain models need to be for the analysis at hand.

The generalizability of Dynamic Sensitivity Analysis to a wide range of modelling works, allows exploring distinct ‘control’ features that may be needed to switch the brain to a different brain state. In practice, every parameter of the model that has an impact on the brain's spatio-temporal dynamics can be thought of as a control parameter. Depending on their biophysical realism and level of detail, different models will have different parameters, but most will at least contain the global coupling strength, which tunes the level of interactions amongst the regional models, and positions the brain at the optimal dynamic working point. At the “healthy” or optimal working point the dynamics have been shown to exhibit near-critical properties where many of the informational processing features are maximised [103]. At the level of perturbations, when understood what the right perturbation sites and profiles are, one can “control” the optimal perturbation as one knows what the right type of perturbation is to achieve the desired outcome. Then, this choice of perturbation parameters can be thought of as a controlled agent. However, as mentioned in the Introduction, this doesn’t imply that the chosen parameter controls the trajectory to the target states. Rather, the perturbation with these parameters “nudges” the system to the desired outcome state.

In our assumption a balanced brain is an optimal dynamical regime that can be related to a healthy neurotypical brain state. For instance on the macroscopic scale, brains of healthy participants are known to possess well-balanced integrative and segregative tendencies [104], work at a dynamical regime of near-criticality where many of information processing properties are maximised [105] and contain turbulent-like dynamics of maximally efficient information transfer [106]. In altered brain states many of these properties are impaired. So from this angle to “rebalance” suggests to promote dynamic change to that of the spatio-temporal activity associated with healthy neurotypical brain states. In Dynamic Sensitivity Analysis, we, for instance, consider the brain dynamics to be “rebalanced” if they achieve a similar PMS distribution as the control group/condition. However other measures defining the spatiotemporal dynamics of brain states and their probabilities of occurrences could potentially be employed as well, as described earlier in the section ‘Spatiotemporal Dynamics of Brain States’. This optimal dynamical working point is probably well maintained through homeostatic means but no explicit mechanism is assumed in the context of whole-brain models. For example, the hopf model’s intrinsic stimulation (from changes to the bifurcation parameter) leads to rebalancing the dynamics by changing the nature of the local dynamical profile (be it noise or oscillation-driven) this in turn can be related to changes in the local excitatory-inhibitory balanced which is known to be a well-maintained homeostatic process for good functioning of the brain. Alternatively, more explicit examples have shown how a local inhibitory feedback loop can serve as a homeostatic mechanism that maintains a certain dynamical regime. This has been performed for example in eNMM [79] and Wilson-Cowan model [57].

In theory there is no conceptual or methodological issue with being able to use linear models in a whole-brain paradigm. Indeed, there have been several studies applying and fitting linear whole-brain models to the functional MRI signals - multivariate Ornstein-Uhlenbeck process [46] or spatial autoregressive (SAR) models [44], [45] are just two examples. And they might very well be used if one wishes to validate the models to the grand-average functional connectivity or as also known the static FC. Further on this point, SAR models have been shown to, at times, outperform some of the non-linear models [48], however, as we argued above and throughout this review, for an optimal rebalancing of brain states one wishes to have a rich spatio-temporal description of brain states. This we have argued conceptually [103] but also from the ability to predict different brain states [8]. As such it is imperative to move beyond a mere time-collapsed picture of brain activity as signified by static FC and consider other dynamical measures such as the Functional Connectivity Dynamic or Probabilistic Metastable Substates. In such light, linear models might fall short of adequately describing the spatio-temporal signatures as explored in [8], [48] where non-linear models were shown to model FCD spectrum unlike linear models.

## Funding

The authors declare that they have no conflict of interest. J.V. is supported by the EU H2020 FET Proactive project Neurotwin grant agreement no. 101017716. J.C. is funded by the Portuguese Foundation for Science and Technology grants UIDB/50026/2020, UIDP/50026/2020, la Caixa” Foundation (LCF/BQ/PR22/11920014) and CEECIND/ 03325/2017, Portugal. F.C. is funded by the EU-project euSNN European School of Network Neuroscience (MSCA-ITN-ETN H2020-860563). The Wellcome Centre for Human Neuroimaging is supported by core funding from Wellcome [203147/Z/16/Z]. M.L.K. is supported by the Center for Music in the Brain, funded by the Danish National Research Foundation (DNRF117), and Centre for Eudaimonia and Human Flourishing at Linacre College funded by the Pettit and Carlsberg Foundations. G.D. is supported by the Spanish national research project (AEI-PID2019-105772GB I00/AEI/10.13039 /501100011033) funded by the Spanish Ministry of Science, Innovation and Universities (MCIU), State Research Agency (AEI).

## Declaration of Competing Interest

The authors declare that they have no known competing financial interests or personal relationships that could have appeared to influence the work reported in this paper.
